# Broken Heart: A Clear Case of Takotsubo Cardiomyopathy

**DOI:** 10.7759/cureus.48685

**Published:** 2023-11-12

**Authors:** Mohamed A Ibrahim, Moayad A Elgassim, Amro Abdelrahman, Wala Sati, Hany A Zaki, Mohamed Elgassim

**Affiliations:** 1 Emergency Department, Hamad Medical Corporation, Doha, QAT; 2 School of Medicine, Taylor's University Lakeside Campus, Subang Jaya, MYS; 3 Medical Education Department, Hamad Medical Corporation, Doha, QAT

**Keywords:** high troponin-t, lv ejection fraction (lvef), ekg abnormalities, cardio, tako-tsubo syndrome

## Abstract

Takotsubo cardiomyopathy (TC) is a recognized clinical syndrome characterized by reversible cardiomyopathy with a distinctive left ventricular apical ballooning appearance. TC is associated with risk factors such as estrogen deficiency, emotional and physical stress, and genetic factors. The clinical presentation of TC can be like that of a myocardial infarction. While catecholamine-induced myocardial stunning is suggested by current evidence, the exact pathophysiological mechanisms remain uncertain. Diagnostic criteria, including the InterTAK Diagnostic Criteria, have been established by the Takotsubo International Registry. Supportive and symptomatic medication constitutes the mainstay of treatment, with a focus on improving left ventricle (LV) function over several days, leading to full recovery within three to four weeks. Given its resemblance to myocardial infarction, cautious diagnosis and management are essential for optimal outcomes. We present the case of a previously healthy 35-year-old female who presented with chest pain and dyspnea after discovering her father's death. On examination, she exhibited hypotension, bradycardia, and a new-onset left bundle branch block (LBBB) in her electrocardiogram. Her left ventricular ejection fraction (LVEF) on presentation was 22%, and troponin T (TnT) levels were notably elevated at 430 (normal ranges < 14). After two days of treatment and monitoring at the cardiac intensive care unit (CICU), she improved clinically, and her LVEF improved to 52%.

## Introduction

Takotsubo cardiomyopathy, also known as a broken-heart syndrome or apical ballooning syndrome, is a transient, reversible left ventricular (LV) systolic dysfunction after intense emotional or physical stress in the absence of obstructive evidence of coronary artery disease. Takotsubo is a pot that the Japanese people traditionally used to catch octopus. In this condition, the shape of the heart resembles this pot [1]. Typically, the affected patient is a postmenopausal woman [2]. The disease presents in a similar fashion to acute coronary syndrome (ACS). About 2% of ACS cases have been linked to Takotsubo cardiomyopathy [1], so recognizing the disease is very important in order to provide proper management for the affected population.

## Case presentation

**Figure 1:**
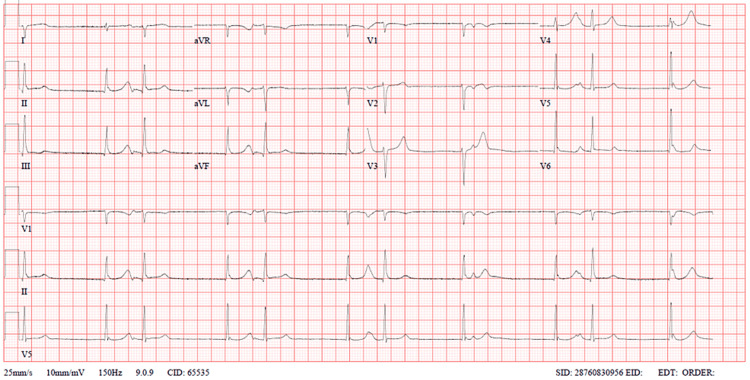
ECG showing junctional rhythms

**Figure 2:**
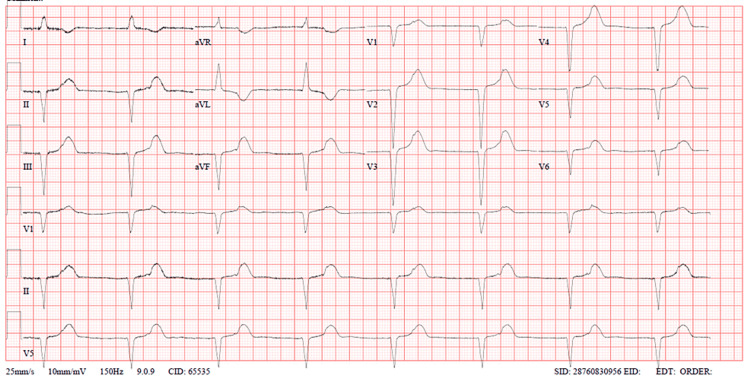
ECG showing new onset LBBB

**Figure 3:**
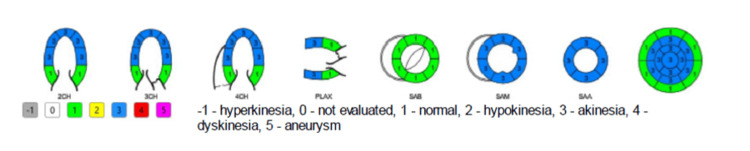
Echocardiography report The following diagram demonstrates an echocardiogram. It demonstrates regional wall abnormalities in the heart. Wherein the kinetic ability of the heart region is numerated and colored coded, -1 is hyperkinetic and grey, 0 is not evaluated and white, 1 is normal and green, 2 is hypokinesia and yellow, 3 is akinesia and blue, 4 is dyskinesia and red, and 5 is aneurysm and pink. 2CH is 2 chamber view, 3CH is 3 chamber view, 4CH is 4 chamber view, PLAX is long access view, SAB is short access mitral valve, SAM is short access papillary muscles, and SAA is short access apical.

We present the case of a 35-year-old lady who presented to the emergency department with a sudden onset of chest pain one hour before arrival. The onset of symptoms was preceded by her receiving the news of the death of her father. She has no background history of chronic medical illness or surgeries, except for a history of bradycardia two years before her presentation. Upon presentation, the patient was in severe chest pain that was radiating to her shoulders and back, which was associated with shortness of breath, headache, nausea, and one episode of vomiting. On initial triage, she was found to be sick-looking; her vitals showed bradycardia of 50 bpm and hypotension of 78/62 mmHg. The initial electrocardiogram (ECG) (Figure 1) showed junctional rhythms. The patient was immediately shifted to the resuscitation area for monitoring and further care. After 45 minutes, the patient was hypoxic at 92% on room air, and her hypotension worsened; thus, her ECG was repeated (Figure 2), showing a new-onset left bundle branch block (LBBB). Her bedside point-of-care ultrasound (POCUS) showed a severely reduced ejection fraction (EF) of 22% with poor contractility except at the bases (Figure 3, Videos 1, 2). A chest x-ray showed signs of pulmonary edema.

**Video 1:** Long axis ultrasound view

**Video 2:** Four chamber ultrasound view

**Figure 4:**
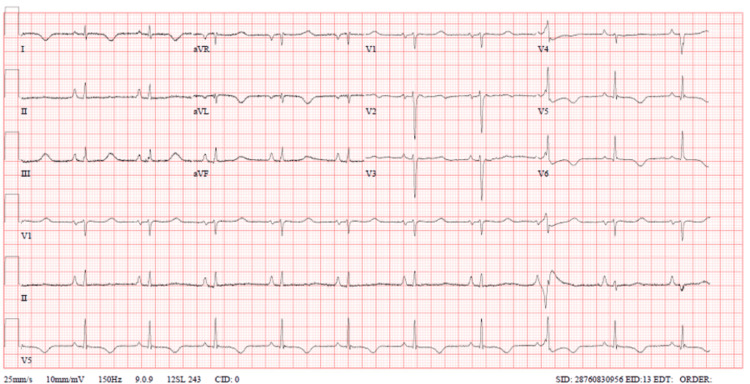
Resolution of LBBB

The patient was started on furosemide and noradrenaline and slowly started to improve. The first troponin T (TnT) came back positive at 430. Cardiology was consulted for the patient, and it was decided to admit the patient to the coronary intensive care unit (CICU) unit as a case of cardiogenic shock due to Takotsubo disease and start her on Aspirin 300mg, clopidogrel 300mg, and heparin 4,000 units IV. A repeat ECG (Figure 4) showed the resolution of LBBB; however, it also showed fusion complexes and right atrial enlargement. In the CICU, dobutamine was added to the management, and a transvenous pacing wire was inserted as she has a junctional rhythm and was kept on standby in case of deterioration. She was kept on hemodynamically guided management, requiring an intra-aortic balloon pump (IABP) and intravenous (IV) diuresis. Two days after continuous monitoring in the CICU, she was weaned off noradrenaline, and the transvenous pacemaker and IABP were removed. On day 4, she was weaned off dobutamine, and the patient was transferred to the ward. Albeit delayed, coronary angiography was eventually done and revealed normal coronaries. In the ward, the patient’s echocardiogram revealed left ventricle ejection fraction (LVEF) had improved to 52%, and after a total of 13 days of monitoring, the patient was discharged with a monthly follow-up. The patient remained well afterward.

## Discussion

While the disease has been linked to various psychosocial stress triggers, the precise mechanism behind Takotsubo cardiomyopathy remains not fully understood. One of the most widely acknowledged explanations in the scientific literature relates to the impact of surges in catecholamines subsequent to a stressful event [3]. The elevated levels of catecholamines resulting from stress-induced sympathetic stimulation can have adverse effects on the heart's microvascular circulation, potentially leading to compromised myocardial function [3].

Furthermore, low levels of estrogen have been correlated with the onset of Takotsubo cardiomyopathy in postmenopausal women facing an estrogen deficiency associated with aging. Increased estrogen levels can mitigate the risk of cardiac damage resulting from emotional stress by regulating neurological and cardiac pathways [4]. It is noteworthy that medical conditions such as asthma, diabetes, and serotonin syndrome have been linked to the emergence of Takotsubo cardiomyopathy [5].

Classically, the patient presents with cardiopulmonary symptoms like chest pain, dyspnea, syncope, etc., with a background of a recent stressful event [5]. Hence, it is very beneficial to get a proper history from any suspected female patient presenting with symptoms of ACS. The diagnosis of Takotsubo can be tricky as it presents in a similar fashion to ACS. However, the revised Mayo Clinic diagnostic criteria have provided physicians with the essential tools to diagnose this condition [6].

In this case, the patient presented with chest pain following the recent news of her father's death. Vitally, she was bradycardic and hypotensive. Her initial ECG showed first-degree heart block with supraventricular complex patterns. In the resuscitation area, she was found to have high troponin T, an EF of 22%, new onset LBBB on ECG, and radiographic evidence of pulmonary edema. She was shifted immediately to the CICU as a case of cardiogenic shock due to Takotsubo cardiomyopathy.

In the treatment of Takotsubo cardiomyopathy, the main approach is the management of symptoms. IABP equipment is necessary for hemodynamically unstable patients, as was performed in our patient. She was started on furosemide and noradrenaline, and a transvenous pacing wire was inserted. Patients who experience severe LV outflow tract obstruction and hemodynamic instability may benefit from treatment using beta-blockers or alpha-adrenoceptor agonists [7].

Calcium channel blockers can be used to alleviate vasospasms and reduce the pressure gradient in the LV outflow tract. Despite previous literature stating that nitrated or inotropic drugs should be avoided, our patient was given dobutamine in the CICU and had a good prognosis. The patient was weaned off on the fourth day after the onset of symptoms. Anticoagulation therapy can be considered to reduce the risk of thromboembolism unless there is a definitive contraindication. Our patient was started on Aspirin 300mg, Clopidogrel 300mg, and heparin 4,000 units. Hemodynamically stable patients often receive treatment with beta-blockers, angiotensin-converting enzyme inhibitors, and diuretics. However, there is conflicting evidence regarding the effectiveness of long-term administration of these medications in individuals with Takotsubo cardiomyopathy [8].

After four days, the patient was stepped down to the medical ward after she was stabilized in the CICU. Angiography was done, and the result showed no evidence of coronary obstruction, and her EF improved significantly (from 22% at presentation to 52% in the medical ward). She was discharged with a monthly follow-up with a cardiology clinic.

## Conclusions

Takotsubo cardiomyopathy is a transient and reversible LV systolic dysfunction following intensive emotional or physical stress in the absence of coronary artery disease. We hereby present a case of Takotsubo cardiomyopathy with clear findings in ECG, and echocardiography. Coronary angiography was done and revealed normal coronaries. Takotsubo cardiomyopathy can mimic signs of ACS; therefore, healthcare professionals should be vigilant when considering Takotsubo cardiomyopathy as a potential diagnosis, especially in those with signs of ACS and a history of psychosocial stressors. Prompt recognition and treatment are essential for favorable outcomes.
